# Hydrocortisone treatment is associated with early recovery from severe septic shock in patients with obstructive pyelonephritis due to upper urinary tract stone

**DOI:** 10.1002/bco2.498

**Published:** 2025-02-17

**Authors:** Isamu Otsuka, Koshiro Nishimoto, Taichi Kozako, Katsuhiro Kanemaru, Yasuhiro Yamashita, Toshiyuki Kamoto, Atsuro Sawada

**Affiliations:** ^1^ Department of Urology Miyazaki Prefectural Nobeoka Hospital Miyazaki Japan; ^2^ Department of Urology University of Miyazaki Faculty of Medicine Miyazaki Japan; ^3^ Department of Emergency Medicine Miyazaki Prefectural Nobeoka Hospital Miyazaki Japan

**Keywords:** hydrocortisone, pyelonephritis, septic shock, urinary stone

## Abstract

**Objectives:**

The administration of hydrocortisone in patients with severe septic shock contributes to early recovery in intensive care. The purpose of this study was to evaluate the effect of hydrocortisone on early recovery from severe septic shock in patients with obstructive pyelonephritis due to upper urinary tract stone (stone pyelonephritis).

**Methods:**

From January 2018 to October 2023, of all patients admitted for treatment of stone pyelonephritis, 28 did not respond to initial fluid infusion and vasopressors for urosepsis. Among these 28 patients, 14 were administered hydrocortisone for recovery from early shock. Characteristics and noradrenaline administration time of patients treated or not treated with hydrocortisone were retrospectively analysed.

**Results:**

In patients with septic shock associated with stone pyelonephritis unresponsive to initial fluid and vasopressors, noradrenaline administration time in the hydrocortisone group (28.7 ± 17.5 h) was significantly shorter than in the non‐treated group (46.0 ± 12.8 h, *p* = 0.006). The factors diabetes, blood culture results, age, performance status, severity of vital signs and laboratory data on sepsis severity were not significantly associated with the duration of noradrenaline administration.

**Conclusions:**

Our findings suggest potential benefits of hydrocortisone administration for stone pyelonephritis unresponsive to initial fluid and vasopressors. Widespread adoption of hydrocortisone in the treatment of sepsis, which is common in intensive care, could become more important in urology.

## 1 INTRODUCTION

Acute pyelonephritis following obstruction of urinary stone(s) (stone pyelonephritis) can be lethal if it progresses to severe septic shock.^1^, ^2^ Management of the condition includes treatment for shock (e.g., infusions of noradrenaline and extracellular fluid, haemodialysis) as well as emergent drainage of hydronephrosis through ureteral catheter/stent or nephrostomy. Infusion of noradrenaline intensely constricts peripheral arteries, leading to an increase in blood pressure.^1^ However, prolonged use of noradrenaline can cause diverse organ damage including ischemia of extremities (especially in diabetes patients) and myocardial infarction. It is reported that diabetes, advanced age, performance status (PS), thrombocytopenia, C‐reactive protein (CRP) level and bacteremia are risk factors for mortality in sepsis with obstructive pyelonephritis.^2^, ^3^ However, there are no reports thus far on factors related to the duration of noradrenaline administration.

In recent years, it has been reported that hydrocortisone infusion is effective for patients who have poor response to initial treatment for shock status (i.e., mean arterial pressure <65 mmHg).^4^ The treatment strategy is based on the support of relative adrenal insufficiency due to severe stress via sepsis. The European Association of Urology guideline states that hydrocortisone administration may support the rescue of such patients,^5^ whereas hydrocortisone usage is not mentioned in guidelines of the American Urological Association,^6^ Urological Association of Asia^7^ and Japanese Urological Association. In this study, we took advantage of the fact that many patients with stone pyelonephritis in Japan are not treated with hydrocortisone. We aimed to determine the effectiveness of hydrocortisone by comparing the duration of noradrenaline administration between patients treated with and without hydrocortisone, taking into account several pre‐treatment factors.

## 2 METHODS

### 2.1 Patients and treatments for septic shock

The study was approved by the ethics committee of Miyazaki Prefectural Nobeoka Hospital (approval number: #20240124–1). We retrospectively reviewed 28 patients treated from January 2018 to October 2023 in Miyazaki Prefectural Nobeoka Hospital (*n* = 20) and its affiliated Miyazaki University Hospital (*n* = 8) who survived septic shock following stone pyelonephritis. Vasopressor was initiated if patients' systolic blood pressure was less than 65 mmHg. Sepsis was diagnosed if the quick sequential organ failure assessment score was more than 2.^8^ If the patient did not respond well to initial infusions and vasopressors, they were admitted to the intensive care unit. The use of hydrocortisone depended on the decision of each physician involved. All patients were treated with noradrenaline until recovery from shock (i.e., mean arterial pressure >65 mmHg), and hydrocortisone was initiated within 1 h after the commencement of noradrenaline if it was used. Drainage by creation of nephrostomy or replacement of ureteral catheter/stent was performed for all patients immediately after the diagnosis of stone pyelonephritis. The dose of hydrocortisone was 100 mg initially followed by 200 mg per day continuously.

### 2.2 Statistical analyses

Statistical analyses were performed using Python software (version 3.10). Clinical data were analysed using the Shapiro–Wilk test for assessing the distribution of continuous values, Student's *t*‐test, Pearson's correlation coefficient, or Spearman's correlation coefficient. *p* < 0.05 was considered significant.

### 2.3 Results

**FIGURE 1:**
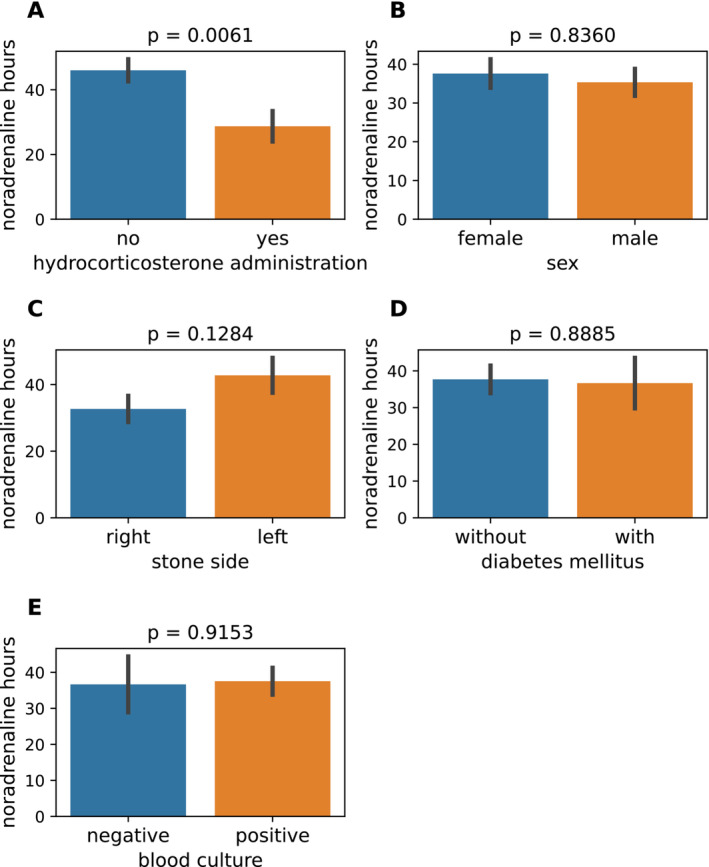
Hydrocortisone administration (A) showed a significant association with noradrenaline hours, as determined by Student's *t*‐test. By contrast, sex (B), side of stone(s) (C), presence of diabetes (D) and blood culture results (E) did not exhibit any significant relationship with noradrenaline hours.

Table 1 shows the patients' characteristics. Two patients underwent the creation of a nephrostomy, while 26 patients underwent replacement of a ureteral catheter/stent. Student's *t*‐tests were performed to determine the association between noradrenaline duration (nearly equal to the shock period) and the two groups of data. As expected, many patients (*n* = 14, 50.0%) did not receive hydrocortisone (“no” in Figure 1A) and the other 14 patients (50.0%) did (“yes” in Figure 1A). Noradrenaline duration in the hydrocortisone‐treated group (28.7 ± 17.5 h [mean±SD]) was significantly shorter than in the non‐treated group (46.0 ± 12.8 h, *p* = 0.006). However, sex (female [37.6 ± 18.4 h] vs. male [35.3 ± 6 h], Figure 1B), side of stone(s) (right [32.7 ± 15.2 h] vs. left [42.8 ± 18.8 h], Figure 1C), and with (36.7 ± 20.6 h) or without (37.7 ± 16.3 h) diabetes mellitus (Figure 1D) showed no significant differences in the noradrenaline hours (*p* = 0.8360, 0.1284 and 0.8885, respectively). Intriguingly, no significant difference was shown in noradrenergic time between cases with negative and positive blood culture (37.7 ± 19 h vs. 37.5 ± 17.5 h, *p* = 0.9153, Figure 1E). Thus, administration of hydrocortisone was possibly associated with a shortened shock time.

**TABLE 1:** Patient characteristics.

Characteristics	All patients (*n* = 28)	Noradrenaline only (*n* = 14)	Hydrocortisone treatment (*n* = 14)	*p* value
Sex, male, *n* (%)	3 (10.7)	1 (7.1)	2 (14.3)	1
Age (years), mean ± SD	78.39 ± 10.7	78.7 ±9.4	78.1 ±12.3	0.8772
ECOG PS, median (IQR)	1 (0–2)	1 (0–2)	0 (0–1)	0.1792
Charlson Comorbidity Index, median (IQR)	2 (1–3)	1.5 (1–2)	2 (1–3)	0.4634
Diabetes mellitus, *n* (%)	9 (32.1)	4 (28.6)	5 (35.7)	1
Stone side, left, *n* (%)	13 (46.4)	10 (71.4)	3 (21.4)	0.0021
Drainage, nephrostomy, *n* (%)	2 (7.1)	1 (7.1)	1 (7.1)	1
BMI (kg/m^2^), mean ± SD	22.63 ± 3.77	20.9 ±3.3	24.4 ±3.5	0.0116
Body temperature (°C), mean ± SD	37.83 ± 1.18	38.0 ±1.3	37.7 ±1.1	0.5324
Mean arterial pressure (mmHg), mean ± SD	62.81 ± 13.27	68.0 ±12.0	57.6 ±12.8	0.0367
Heart rate (/min), mean ± SD	105.00 ± 15.5	100.9 ±11.6	109.1 ±18.1	0.1613
Respiratory rate (/min), mean ± SD	23.25 ± 5.33	23.1 ±5.6	23.4 ±5.3	0.8632
Blood culture, positive, *n* (%)	22 (78.6)	12 (85.7)	10 (71.4)	0.6483
WBC (/μL), mean ± SD	16 914 ± 11 220	13 629 ±12 160	20 200 ±9506	0.1232
CRP (mg/dL), mean ± SD	17.82 ± 10.69	15.3 ±9.6	20.3 ±11.4	0.2203
Procalcitonin (ng/mL), mean ± SD	104.19 ± 83.02	72.4 ±62.7	130.7 ±90.8	0.1021
Platelet (/μL), mean ± SD	93 892 ± 64 875	87 928 ±54 898	99 857 ±75 174	0.6356
Na (mEq/L), mean ± SD	137.54 ± 4.68	138.1 ±5.0	136.9 ±4.4	0.5026
K (mEq/L), mean ± SD	3.89 ± 0.63	3.7 ±0.5	4.0 ±0.7	0.1869
Creatinine (mg/dL), median (IQR)	1.8 (1.5–2.5)	1.7 (1.2–2.0)	2.2 (1.7–2.7)	0.1078
Total bilirubin (mg/dL), median (IQR)	0.94 (0.62–1.42)	1.1 (0.7–1.4)	0.8 (0.6–1.2)	0.5348
Noradrenaline time (hours), mean ± SD	37.36 ± 17.43	46.0 ±12.8	28.7 ±17.5	0.0061

SD: Standard Deviation, IQR: Interquartile Range, ECOG PS: Eastern Cooperative Oncology Group Performance Status, BMI: Body Mass Index, WBC: White Blood Cell, CRP: C‐Reactive Protein.

**FIGURE 2:**
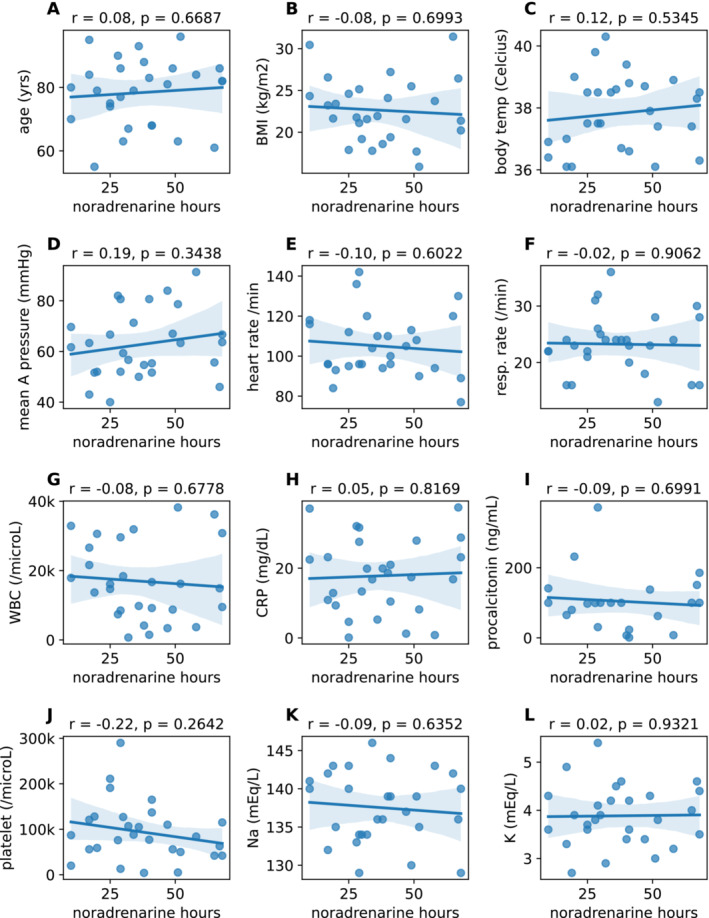
Age (A) and body mass index (BMI, B) demonstrated no significant correlation with noradrenaline hours based on Pearson's correlation coefficient test. Similarly, vital signs (C–F) and laboratory data (G–L) also did not show any significant correlations.

Normality tests showed that 12 items had normal distributions (Figure S1). Mean age and noradrenaline hours were 78.4 ± 10.7 years and 37.4 ± 17.4 h, respectively (Figure 2A). No significant correlation was observed between age and noradrenaline hours (*r* = 0.08, *p* = 0.6687, Pearson's correlation coefficient). Similarly, body mass index (BMI) was 22.6 ± 3.8 kg/m^2^ and showed no correlation with noradrenaline hours (*r* = −0.08, *p* = 0.6993, Figure 2B). Against our expectations, we discovered that parameters generally used for assessing the severity of vital signs, namely body temperature (37.8°C ± 1.18°C), mean arterial pressure (62.8 ± 13.3 mmHg), heart rate (105 ± 16/min) and respiratory rate (23 ± 5/min), were not significantly associated with noradrenaline hours (*r* = 0.12, 0.19, −0.10 and −0.02, respectively; *p* = 0.5345, 0.3438, 0.6022 and 0.9062, respectively; Figure 2C–F). Similarly, laboratory data associated with infection and inflammation, namely white blood cell (WBC) count (16 914 ± 11 220/μL), CRP (17.8 ± 10.7 mg/dl), procalcitonin (104.2 ± 83 ng/ml) and platelet count (97 893 ± 64 876/μL), did not show a significant association with noradrenaline hours (*r* = −0.08, 0.05, −0.09 and −0.22, respectively; *p* = 0.678, 0.817, 0.699 and 0.264, respectively; Figure 2G–J). Electrolytes serum sodium (Na, 138 ± 5 mEq/l) and potassium (K, 3.9 ± 0.6 mEq/l) were likewise not associated with noradrenaline hours (*r* = −0.09 and 0.02, respectively; *p* = 0.635 and 0.932, respectively; Figure 2K,L). It was especially noteworthy that, like the existence of bacteria in blood (Figure 1E), severity of vital signs (Figure 2C–F) and laboratory data on severity of sepsis (Figure 2G–L) were not associated with noradrenaline hours.

**FIGURE 3:**
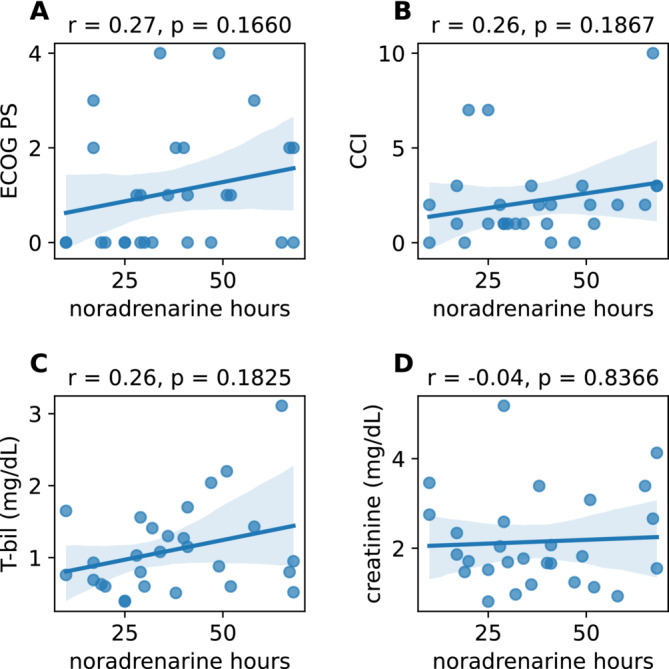
Eastern Cooperative Oncology Group performance status (ECOG PS, A) and Charlson Comorbidity Index (CCI, B) did not correlate significantly with noradrenaline hours as assessed by Spearman's correlation coefficient test. Additionally, specific laboratory data (C and D) showed no significant association with noradrenaline hours.

Normality tests showed that four items had a non‐normal distribution (Figure S1). The median PS and Charlson Comorbidity Index (CCI) were 1.0 (interquartile range [IQR] 0.0–4.0, Figure 3A) and 2 (IQR 0–10, Figure 3B), respectively. PS and CCI were not significantly correlated with noradrenaline hours (*r* = 0.27 and 0.26, respectively; *p* = 0.166 and 0.187, respectively; Spearman's correlation coefficient). Similarly, median serum creatinine of 1.79 mg/dl (IQR 0.81–5.18 mg/dl, Figure 3C) and serum total bilirubin of 0.94 mg/dl (IQR 0.39–3.11 mg/dl) were not significantly correlated with noradrenaline hours (*r* = 0.26 and −0.04, respectively; *p* = 0.183 and 0.837, respectively). Overall, with the exception of hydrocortisone administration (Figure 1A), sex (Figure 1B), side of stone(s) (Figure 1C), absence or presence of diabetes (Figure 1D), blood culture (Figure 1E), age (Figure 2A), BMI (Figure 2B), vital signs (Figure 2C– F), laboratory data (Figures 2G–L and 3C,D), PS (Figure 3A) and CCI (Figure 3B) showed no association with noradrenaline hours.

## 3 DISCUSSION

In this study, we analysed the duration of noradrenaline administration for septic shock in patients with obstructive pyelonephritis and upper urinary tract stone(s). In patients with septic shock associated with pyelonephritis treated with hydrocortisone when unresponsive to initial fluid and vasopressors, the noradrenaline duration in the hydrocortisone group (28.7 ± 17.5 h) was significantly shorter than in the non‐treated group (46.0 ± 12.8 h, *p* = 0.006).

Cortisol (also known as hydrocortisone) is the major corticosteroid secreted by upregulation of the hypothalamic–pituitary‐adrenocortical (HPA) axis.^9^ In septic patients the HPA axis is often impaired, leading to relative adrenal insufficiency termed critical illness‐related corticosteroid insufficiency (CIRCI).^10^, ^11^ CIRCI is characterized by inadequate cortisol production and decreased glucocorticoid receptor function, contributing to hemodynamic instability. The adrenocorticotropic hormone (ACTH) stimulation test was previously performed to evaluate CIRCI, whereby patients were diagnosed with CIRCI if their cortisol level was below 9 μg/dl even after ACTH stimulation.^12^ Therefore, the administration of hydrocortisone may aid recovery in patients with severe shock.

Based on the concept of CIRCI, several clinical trials have been conducted. In the corticosteroid therapy of septic shock (CORTICUS) trial, a phase 3 clinical trial, patients administered hydrocortisone showed faster recovery than those administered placebo.^13^ Other phase 3 trials, the FRENCH trial14 and the ADRENAL trial,^4^ supported this finding. Based on these results, practice guidelines for corticosteroid therapy for sepsis^14^ and the international guidelines for the management of sepsis and septic shock^15^ recommend the use of hydrocortisone for CIRCI if the initial infusion of vasopressors is not sufficiently effective. In addition, these trials primarily focused on septic shock across various organ systems, with the proportion of cases attributable to urological causes ranging from 4.7% to 7.5%.^4^, ^16^ As urological septic shock constitutes a relatively small subset in these studies, its unique characteristics and management strategies remain underexplored. To our knowledge, this study is the first to specifically investigate the effects of hydrocortisone in urological septic shock, representing a novel and valuable contribution to the literature. Although the current study was performed using a small number of patients owing to the difficulty of collecting many cases, our results suggest that hydrocortisone administration may be effective in septic shock caused by stone pyelonephritis, consistent with results from the aforementioned trials.

## AUTHOR CONTRIBUTIONS

**Isamu Otsuka**: Conceptualization; writing—original draft; data curation; formal analysis. **Koshiro Nishimoto**: Writing—original draft; writing—review and editing. **Taichi Kozako**: Data curation. **Katsuhiro Kanemaru**: Supervision; project administration. **Yasuhiro Yamashita**: Supervision; project administration. **Toshiyuki Kamoto**: Supervision; project administration. **Atsuro Sawada**: Writing—original draft; writing—review and editing.

## CONFLICT OF INTEREST STATEMENT

Toshiyuki Kamoto is an Editorial Board member of International Journal of Urology and a co‐author of this article. To minimize bias, they were excluded from all editorial decision‐making related to the acceptance of this article for publication.

## APPROVAL OF THE RESEARCH PROTOCOL BY AN INSTITUTIONAL REVIEW BOARD

The study was approved by the ethics committee of Miyazaki Prefectural Nobeoka Hospital (approval number: #20240124‐1).

## INFORMED CONSENT

N/A.

## REGISTRY AND THE REGISTRATION NO. OF THE STUDY/TRIAL

N/A.

## ANIMAL STUDIES

N/A.

## Supporting information


**Figure S1.** Histograms (left column) and quantile‐quantile (Q‐Q) plots (right column) for the following factors are displayed: age (years), body mass index (BMI, kg/m^**2**^), body temperature (°C), mean arterial pressure (mmHg), heart rate (beats per minute), respiratory rate (breaths per minute), white blood cell count (WBC, /μL), C‐reactive protein (CRP, mg/dL), procalcitonin (ng/mL), platelet count (/μL), sodium (Na, mmol/L), potassium (K, mmol/L), performance status (score), creatinine (mg/dL), total bilirubin (mg/dL), Charlson Comorbidity Index (score) and noradrenaline time (hours). In the histograms, the bars represent the frequency of observed values for each factor, while the overlaid curve indicates the kernel density estimate, providing a smoothed approximation of the distribution. The Q‐Q plots assess the normality of these distributions by comparing ordered values to theoretical quantiles.
