# Supplementary material for: Hydrocortisone treatment is associated with early recovery from severe septic shock in patients with obstructive pyelonephritis due to upper urinary tract stone
Source: BJUI Compass. 2025 Feb 17;6(2):e498. doi: 10.1002/bco2.498 (PMC11832297; doi:10.1002/bco2.498)
Supplement: Supplementary file 1 — Figure S1. Histograms (left column) and quantile‐quantile (Q‐Q) plots (right column) for the following factors are displayed: age (years), body mass index (BMI, kg/m 2 ), body temperature (°C), mean arterial pressure (mmHg), heart rate (beats per minute), respiratory rate (breaths per minute), white blood cell count (WBC, /μL), C‐reactive protein (CRP, mg/dL), procalcitonin (ng/mL), platelet count (/μL), sodium (Na, mmol/L), potassium (K, mmol/L), performance status (score), creatinine (mg/dL), total bilirubin (mg/dL), Charlson Comorbidity Index (score) and noradrenaline time (hours). In the histograms, the bars represent the frequency of observed values for each factor, while the overlaid curve indicates the kernel density estimate, providing a smoothed approximation of the distribution. The Q‐Q plots assess the normality of these distributions by comparing ordered values to theoretical quantiles. [file BCO2-6-e498-s001.pdf]

Supplemental Figure 1

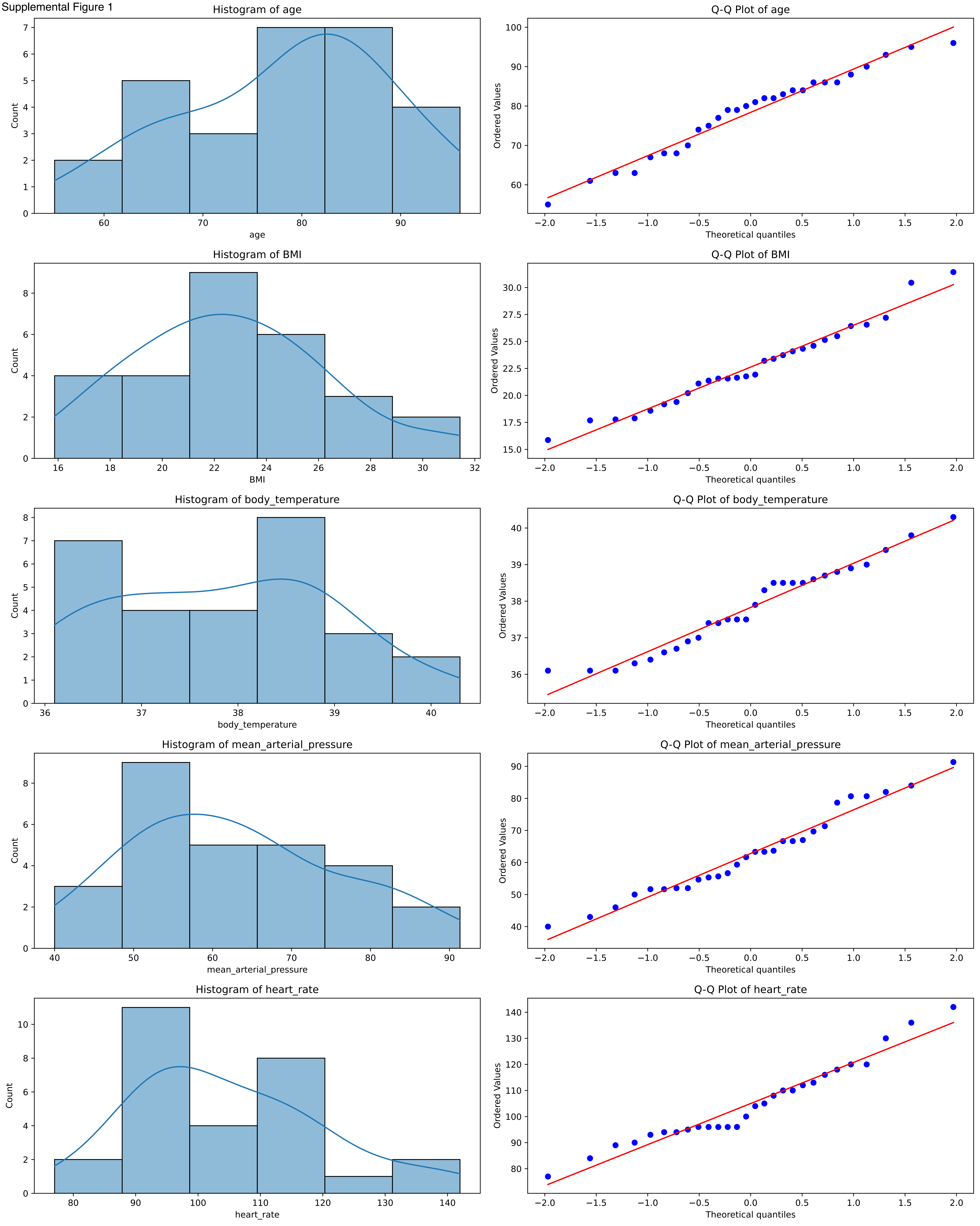

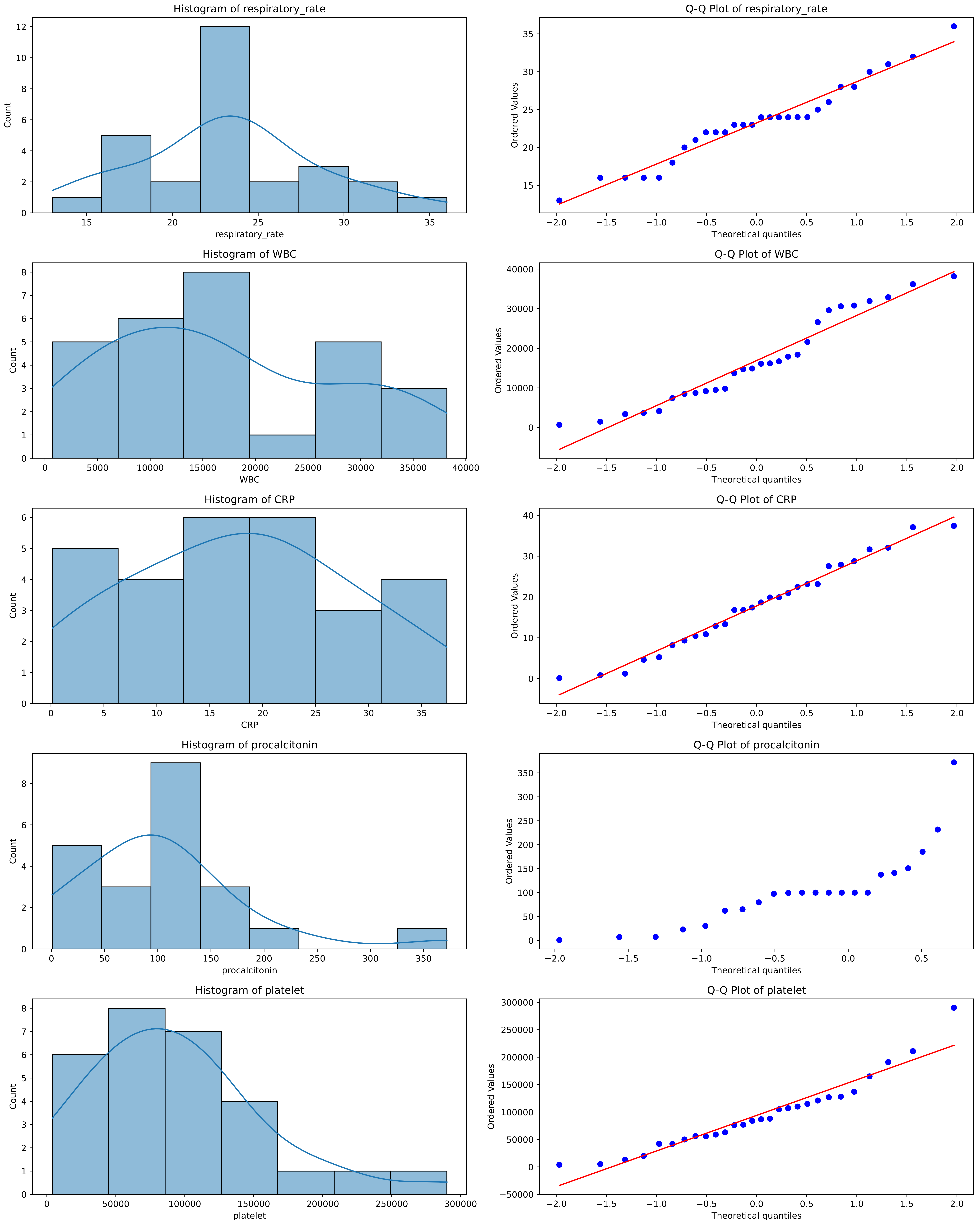
Histogram of respiratory_rate
Count
respiratory_rate
Q-Q Plot of respiratory_rate
Ordered Values
Theoretical quantiles
Histogram of WBC
Count
WBC
Q-Q Plot of WBC
Ordered Values
Theoretical quantiles
Histogram of CRP
Count
CRP
Q-Q Plot of CRP
Ordered Values
Theoretical quantiles
Histogram of procalcitonin
Count
procalcitonin
Q-Q Plot of procalcitonin
Ordered Values
Theoretical quantiles
Histogram of platelet
Count
platelet
Q-Q Plot of platelet
Ordered Values
Theoretical quantiles

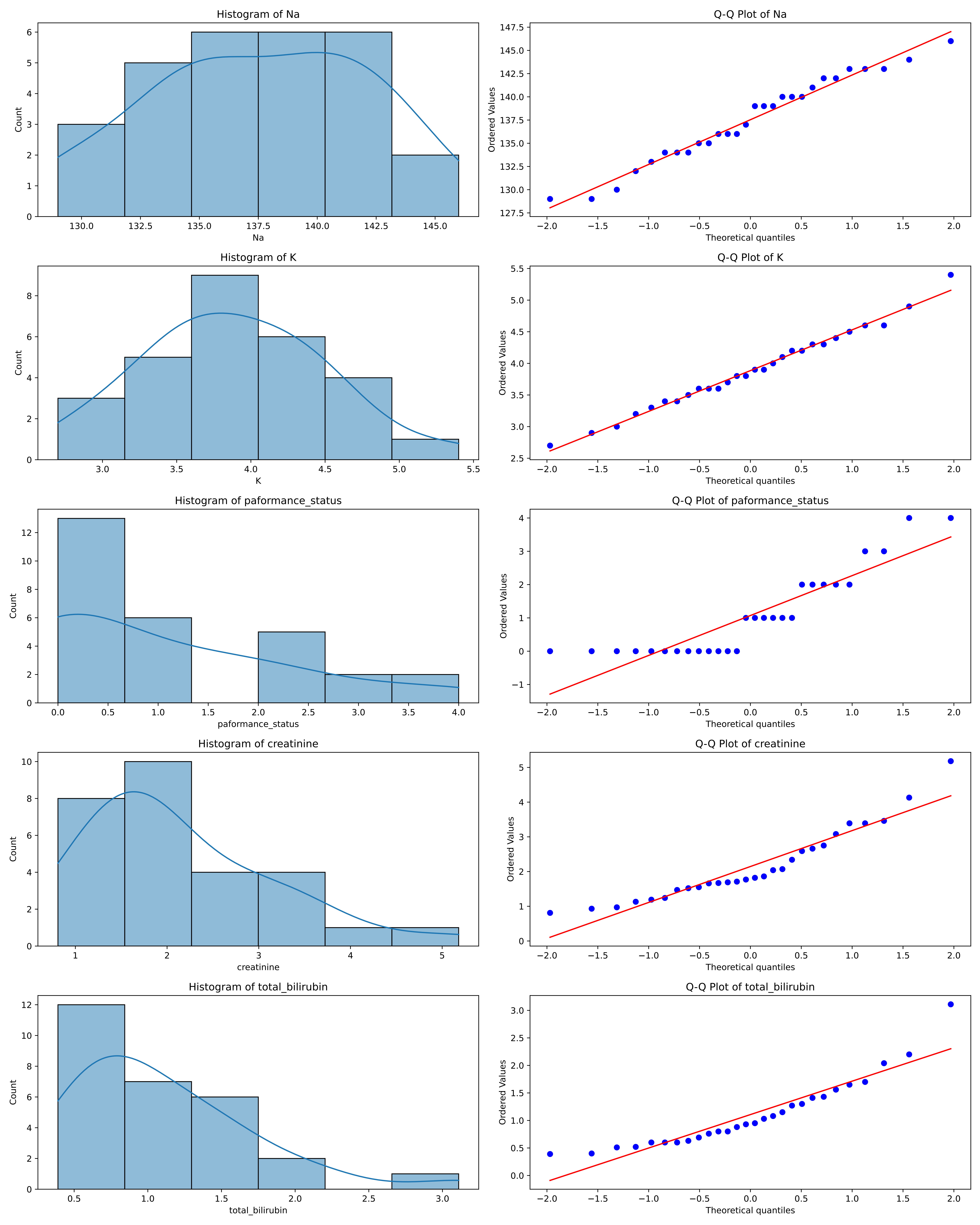
Histogram of Na
Count
Na
Q-Q Plot of Na
Ordered Values
Theoretical quantiles
Histogram of K
Count
K
Q-Q Plot of K
Ordered Values
Theoretical quantiles
Histogram of paformance_status
Count
paformance_status
Q-Q Plot of paformance_status
Ordered Values
Theoretical quantiles
Histogram of creatinine
Count
creatinine
Q-Q Plot of creatinine
Ordered Values
Theoretical quantiles
Histogram of total_bilirubin
Count
total_bilirubin
Q-Q Plot of total_bilirubin
Ordered Values
Theoretical quantiles

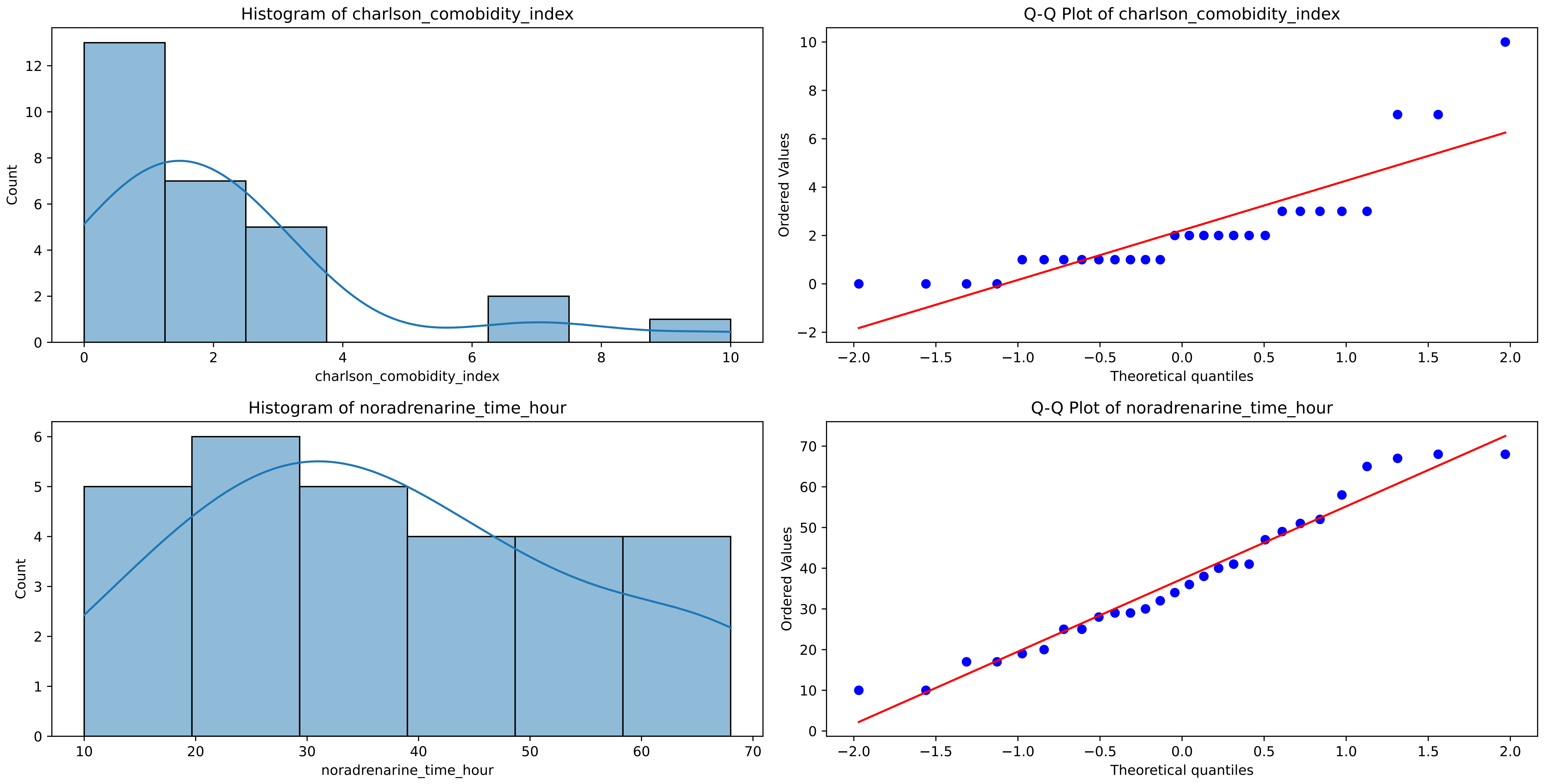
Histogram of charlson_comobidity_index
Count
charlson_comobidity_index
Q-Q Plot of charlson_comobidity_index
Ordered Values
Theoretical quantiles
Histogram of noradrenarine_time_hour
Count
noradrenarine_time_hour
Q-Q Plot of noradrenarine_time_hour
Ordered Values
Theoretical quantiles
